# Infective endocarditis and stroke: when does it bleed? A single center retrospective study

**DOI:** 10.1186/s42466-023-00239-7

**Published:** 2023-04-06

**Authors:** L. Nitsch, O. Shirvani Samani, M. Silaschi, M. Schafigh, S. Zimmer, G. C. Petzold, C. Kindler, F. J. Bode

**Affiliations:** 1 Department of Neurology, University Hospital Bonn, Venusberg-Campus 1, 53127 Bonn, Germany; 2 Division of Vascular Neurology, Department of Neurology, University Hospital Bonn, Venusberg-Campus 1, 53127 Bonn, Germany; 3 Department of Cardiac Surgery, Heart Center Bonn, University Hospital Bonn, Venusberg-Campus 1, 53127 Bonn, Germany; 4 Department of Medicine II, Heart Center Bonn, University Hospital Bonn, Venusberg-Campus 1, 53127 Bonn, Germany; 5 German Center for Neurodegenerative Diseases (DZNE), Venusberg-Campus 1, 53127 Bonn, Germany

**Keywords:** Infective endocarditis, Intracranial hemorrhage, Ischemic stroke, Risk factors, Outcome

## Abstract

**Background:**

Infective endocarditis (IE) is a serious condition with a high mortality, represents a rare cause of stroke and an increased risk of intracranial hemorrhage. In this single center study, we characterize stroke patients with IE. We were interested in risk factors for intracranial hemorrhage and outcome of patients with intracranial hemorrhage compared to patients with ischemic stroke.

**Methods:**

Patients with IE and symptomatic ischemic stroke or intracranial hemorrhage admitted to our hospital between January 2019 and December 2022 were included in this retrospective study.

**Results:**

48 patients with IE and ischemic stroke or intracranial hemorrhage were identified. 37 patients were diagnosed with ischemic stroke, 11 patients were diagnosed with intracranial hemorrhage. The intracranial hemorrhage occurred within the first 12 days after admission. We identified *Staphylococcus aureus* detection and thrombocytopenia as risk factors for hemorrhagic complications. An increased in-hospital mortality in patients with intracranial hemorrhage (63.6% vs. 22%, p = 0.022) was found, whereas patients with ischemic stroke and patients with intracranial hemorrhage do not differ regarding favorable clinical outcome (27% vs. 27.3%, p = 1.0). 27.3% patients with intracranial hemorrhage and 43.2% patients with ischemic stroke underwent cardiac surgery. Overall, 15.7% new ischemic strokes occurred after valve reconstruction, whereas no new intracranial hemorrhage was observed.

**Conclusions:**

We found an increased in-hospital mortality in patients with intracranial hemorrhage. Beside thrombocytopenia, we identified *S. aureus* detection as a risk factor for intracranial hemorrhage.

## Background

Infective endocarditis (IE) is a serious condition and represents a rare cause of stroke. The incidence is approximately 3–10 cases per 100.000 [1]. The modified Duke Criteria considering clinical, imaging, and bacteriological findings are the standard for diagnosis of IE [2].

The treatment of IE includes systemic antibiotic therapy and in about 50% of patients, cardiac surgery. It is indicated in patients with heart failure (HF) due to valve dysfunction, large vegetations with risk for embolism or persistent infection despite antibiotic treatment. Although there have been improvements in diagnostics and therapy, both diagnosis and treatment remain difficult and mortality in IE patients remains high. The in-hospital mortality of IE is approximately 18–25% and 1-year mortality is up to 40% [1].

Patients with neurological complications have worse clinical outcome [3]. Neurologic complications of IE include ischemic stroke and intracranial hemorrhage, infectious intracranial aneurysm (IIA), meningitis, and brain abscesses.

Symptomatic stroke occurs in 35% of patients with IE, and asymptomatic cerebrovascular complications in another 30% [4]. Risk factors for embolism are vegetation size and mobility, mitral valve involvement, and *Staphylococcus aureus* (*S. aureus*) detection [3]. The risk of stroke is highest at time of diagnosis and initiation of antibiotic treatment and decreases after two weeks [2, 5].

The prevalence of intracerebral hemorrhage in IE ranges from 7 to 27% [6]. Intracranial hemorrhage in IE patients can result from primary intracerebral hemorrhage (ICH), hemorrhagic transformation of an ischemic stroke (HT) and subdural hemorrhage or subarachnoid hemorrhage (SAH). The most common cause is HT with 40%, followed by rupture of IIA [7]. Intravenous thrombolysis (IVT) is contraindicated in ischemic stroke patients with IE. However, the diagnosis of IE is often not known on admission and instead established later during the inpatient stay.

In this single center study, we aim to characterize stroke patients with IE admitted to our hospital between January 2019 and December 2022. We were interested in risk factors for intracranial hemorrhage and when it occurs. Furthermore, we discuss optimal timing of cardiac surgery in these patients.

## Material and methods

### Population characteristics

Out of 4962 patients with transitory ischemic attack, symptomatic stroke, or left-sided IE (definite or possible IE according to the modified Duke Criteria and treated for IE) admitted to the Department of Neurology, Cardiology or Cardiac surgery of the University Hospital Bonn between January 2019 and December 2022, we included 48 patients from our database, who met the inclusion criteria (symptomatic acute ischemic stroke or intracranial hemorrhage and IE) in this retrospective study. Patients` data were registered until discharge.

### Data collection

The study was designed as a monocentric retrospective cohort study. Patients` data were retrospectively obtained including age, sex, development of intracranial hemorrhage (defined as SAH, ICH, epidural or subdural hematoma). HT was assessed according to the European Cooperative Acute Stroke Study (ECASS) II classification. Hemorrhagic infarction (HI) 1 was defined as scattered small petechiae, no mass effect, HI2 as confluent petechiae, no mass effect, parenchymatous hematoma (PH) 1 as hemorrhage within infarcted tissue, occupying < 30% without substantive mass effect, PH2 as hematoma occupying 30% or more of the infarcted tissue with obvious mass effect. Only PH1 and PH2 were considered as relevant HT and included in the group of patients with intracranial hemorrhage [8]. Medication including oral anticoagulant (OAC) therapy, anticoagulant therapy, antiplatelet therapy (APT) and/or IVT was registered. Infection control was defined as negative blood culture > 48 h after start of antibiotic therapy and < 48 h before intracranial hemorrhage occurred. Thrombocytopenia was defined as platelet count < 100 × 10^9^/ml (moderate to severe thrombocytopenia). In addition, detection of microbleeds in magnetic resonance imaging (MRI) (> 1 T2* hypointensities with a diameter ≤ 10 mm), severe valve regurgitation, mitral valve endocarditis, *S. aureus* detection, the modified Rankin Scale (mRS) premorbid, on admission and discharge, the length of hospital stay and whether the patients underwent cardiac surgery/ valve replacement were registered. Data collection was approved by the local ethics committee of the University of Bonn (303/22 EC University of Bonn).

### Statistical analysis

Data distribution was analyzed with 1-sample Shapiro- Wilk test. The Fisher’s exact test was performed for dichotomic distributed data, the unpaired t-test, or the Mann–Whitney-U-Test for continuous variables. Univariate logistic regression analysis was performed with variables we consider to be independent predictors of intracranial hemorrhage. With multivariate binomial logistic regression analyses, correlation between female gender, hospital stay, thrombocytopenia, IVT, detection of *S. aureus* and incidence of intracranial hemorrhage was analyzed. A p-value of < 0.05 was considered to be statistically significant. For processing raw data and for statistical analysis the GraphPad Prism^®^ program v. 7.05 (GraphPad Software, Inc., La Jolla, CA, USA) was used.

## Results

### Demographics and clinical characteristics

48 patients with ischemic stroke/intracranial hemorrhage and IE were included in this study with a mean age 64.9 ± 13.94 years. Individual characteristics of patients with intracranial hemorrhage are displayed in Table 1. The characteristics of all patients (ischemic stroke and intracranial hemorrhage) are presented in Table 2.

**Table 1:** Characteristics of patients with intracranial hemorrhage

	Age (years)	Sex	Hemorrhage	APT, IVT, AC, T < 24 h	Days after admission	Days after start Ab	Infection control	Premorbid mRS	Admission mRS	Discharge mRS	Hospital stay (weeks)	Surgery (days after admission)
1	74	M	HT (PH2)	APT	11	11	NA	1	3	3	10	Yes (190)
2	72	M	HT (PH1)	AC	0	Not startet	No	0	2	1	6	Yes (73)
3	58	M	Primary ICH	T	0	Not startet	No	0	2	6	2	No
4	42	F	HT (PH1)	T	0	Not startet	No	0	4	1	8	No
5	71	M	SAH	IVT/ T	1	Not startet	No	0	2	1	6	No
6	74	M	SAH	T/APT	1	1	No	0	1	6	1	Yes (7)
7	72	F	HT (PH1)	IVT	1	Not startet	No	?	5	6	2.5	No
8	90	M	Primary ICH	APT	12	9	Yes	4	4	6	4	No
9	66	M	HT (PH2)	AC/T	7	7	No	1	4	6	2	No
10	26	M	SAH, primary ICH, IIA	T	3	3	No	1	4	6	2	No
11	69	M	SAH, HT (PH2)	AC/T	0	Not startet	No	2	5	6	0.2	No

Age, sex, type of hemorrhage, when it occurred, premorbid/admission/discharge mRS, hospital stay, cardiac surgery performed/ not performed and time of surgery are shown for each patient with intracranial hemorrhage. APT, IVT, AC or T in the last 24 h before intracranial hemorrhage, time of intracranial hemorrhage after start of antibiotic therapy and infection control (defined as negative blood culture > 48 h after start of antibiotic therapy and < 48 h before intracranial hemorrhage occurred) are depicted

*Ab* antibiotic therapy, *AC* anticoagulation, *APT* antiplatelet therapy, *ICH* intracranial hemorrhage, *IVT* intravenous thrombolysis, *HT* hemorrhagic transformation of ischemic stroke, *IIA* intracranial infectious aneurysm, *PH* parenchymatous hematoma, *SAH* subarachnoidal hemorrhage, *T* thrombocytopenia

**Table 2:** Baseline characteristics, all patients

	All (n = 48)	Ischemic stroke (n = 37)	Intracranial hemorrhage (n = 11)	P-value
Age, mean (range)	64.90 ± 13.94 (26–90)	64.89 ± 13.01 (39–88)	64.91 ± 17.43 (26–90)	0.997
Female, % (n)	39.6 (19)	45.9 (17)	18.2 (2)	0.161
Hospital stay, weeks, mean (range)	15.66 ± 18.30 (1–90)	18.05 ± 20.02 (1–90)	7.59 ± 6.27 (1–21)	0.264
mRS premorbid				
mRS 0–2, % (n)	83.0 (39/47)	81.1 (30/37)	90.0 (9/10)	0.667
mRS 3–5, % (n)	17.0 (8/47)	18.9 (7/37)	10.0 (1/10)	0.667
mRS admission				
mRS 0–2, % (n)	27.1 (13/48)	24.3 (9/37)	36.4 (4/11)	0.458
mRS 3–5, % (n)	72.9 (35/48)	75.7 (28/37)	63.6 (7/11)	0.458
mRS discharge				
mRS 0–2, % (n)	27.1 (13/48)	27.0 (10/37)	27.3 (3/11)	1.000
mRS 3–5, % (n)	41.7 (20/48)	51.4 (19/37)	9.1 (1/11)	0.016*
In-hospital mortality, % (n)	31.3 (15)	21.6 (8)	63.6 (7)	0.022*
APT on admission, % (n)	31.3 (15)	32.4 (12)	27.3 (3)	1.000
OAC on admission, % (n)	33.3 (16)	35.1 (13)	27.3 (3)	0.729
Thrombocytopenia, % (n)	29.8 (14/47)	18.9 (7/36)	63.6 (7/11)	0.009*
IVT, % (n)	6.3 (3)	2.7 (1)	18.2 (2)	0.127
ECASS II				
HT1	11.4 (5/44)	15.2 (5/33)	0	–
HT2	9.1 (4/44)	12.1 (4/33)	0	–
PH1	6.8 (3/44)	0	27.3 (3/11)	–
PH2	6.8 (3/44)	0	27.3 (3/11)	–
Stroke > 1 cerebral flow area, % (n)	56.3 (27)	51.4 (19)	72.7 (8)	0.304
MRI available, % (n)	45.8 (22)	43.2 (16)	54.5 (6)	–
Microbleeds (adjusted for MRI), % (n)	50.0 (11/22)	43.8 (7/16)	66.7 (4/6)	0.635
*Staphylococcus aureus* detection, % (n)	20.8 (10)	13.5 (5)	45.5 (5)	0.036*
Mitral valve, % (n)	47.9 (23)	43.2 (16)	63.6 (7)	0.311
Severe valve regurgitation (> II°), % (n)	36.2 (17/47)	35.1 (13/37)	40.0 (4/10)	1.000
Indication for surgery	62.5 (30)	64.8 (24)	54.5 (6)	–
Surgery performed, % (n)	39.6 (19)	43.2 (16)	27.3 (3)	0.488
Ischemic stroke after surgery, % (n)	15.7 (3/19)	18.8 (3/16)	0 (0/3)	–
Intracranial hemorrhage after surgery, % (n)	0 (0/19)	0 (0/16)	0 (0/3)	–

*APT* antiplatelet therapy, *ECASS II* European Cooperative Acute Stroke Study II classification, *HI* hemorrhagic infarction, *IVT* intravenous thrombolysis, *MRI* magnetic resonance imaging, *mRS* Modified Rankin Scale, *OAC* oral anticoagulation, *PH* parenchymatous hematoma

78% (n = 37) had an ischemic stroke, 22% (n = 11) were diagnosed with intracranial hemorrhage between day 0–12 after admission. Five patients developed HT (three patients were characterized as PH1, two patients as PH2), two patients primary ICH, two patients SAH, one patient SAH/ HT (PH2) and one patient primary ICH with IIA (Table 1). Patients with intracranial hemorrhage received antibiotic therapy for 0–9 days before the event occurred. Only one of the patients (1/10) with intracranial hemorrhage showed infection control at time of intracranial hemorrhage.

There was no difference in age, sex, length of hospital stay, premorbid mRS, mRS on admission between patients with and without intracranial hemorrhage (Table 2).

All patients with intracranial hemorrhage had received (oral) anticoagulation, APT, IVT or had thrombocytopenia. However, patients with and without hemorrhage complications differed only significantly regarding thrombocytopenia (7/37 vs. 7/11, p = 0.009). Furthermore, detection of *S. aureus* was increased in patients with intracranial hemorrhage (5/37 vs. 5/11, p = 0.036).

**Fig. 1:**
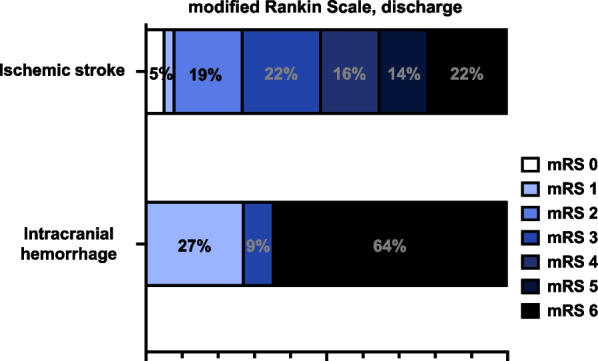
Modified Ranking Scale on discharge. Modified Ranking Scale (mRS) on discharge of patients with ischemic stroke and patients with intracranial hemorrhage

The in-hospital mortality for patients with hemorrhage was significantly increased (8/37 vs. 7/11, p = 0.022) (Table 2, Fig. 1), whereas more patients with ischemic stroke were discharged with mRS 4–5 (11/37 vs. 0//11, p = 0.048). 27.1% patients (13/48) had a favorable outcome (mRS 0–2) on discharge. Patients with good outcome did not differ significantly between the two groups.

All other characteristics including microbleeds (MRI was only in 22 patients, 48%, available), mitral valve endocarditis, severe valve regurgitation or surgery performed showed no significant findings between the two cohorts.

62.5% (n = 30) patients had indication for cardiac surgery, but due to stroke or missing consent of the patient, cardiac surgery was postponed and not performed in several patients. It was performed in 27.3% (n = 3) patients with intracranial hemorrhage (90 ± 92.7 days after admission) and 43.2% (n = 16) patients with ischemic stroke (43.1 ± 50.3 days after event). Overall, 15.7% new ischemic strokes after valve reconstruction were observed, whereas no new intracranial hemorrhage occurred. In all patients without cardiac surgery, the effect of antibiotic therapy was monitored with blood cultures and transesophageal echocardiography.

### Uni- and multivariate binomial logistic regression analyses

Univariable conditional logistic showed that patients with intracranial hemorrhage had more frequently thrombocytopenia (OR 7.556, CI 1.09–52.357, p = 0.041). The other variables age, female, gender, hospital stay, APT on admission, intravenous thrombolysis, stroke > 1 cerebral flow area, microbleeds (adjusted for MRI), mitral valve endocarditis or severe valve regurgitation (> II°) were not predictive for intracranial hemorrhage (Table 3). With multivariate binomial logistic regression analysis, correlation between female gender, hospital stay, thrombocytopenia, IVT, detection of *S. aureus* and incidence of intracranial hemorrhage was analyzed (Table 4). Only thrombocytopenia was a significant predictor of intracerebral hemorrhagic complications (OR 6.5, 95% CI 1.122–49.06, p = 0.045), while the other variables showed no significant effects.

**Table 3:** Intracranial hemorrhage, univariate logistic regression analysis

	OR	95% CI	P-value
Age	1.010	0.946–1.077	0.775
Female	0.400	0.062–2.568	0.334
Hospital stay	0.885	0.700–1.120	0.310
APT on admission	1.600	0.223–11.498	0.640
Thrombocytopenia	7.556	1.090–52.357	0.041*
IVT	7.6	0.567–101.787	0.126
Stroke > 1 cerebral flow area	4.643	0.709–30.418	0.109
MRI performed and available	4.643	0.709–30.418	0.109
Microbleeds (adjusted for MRI)	3.000	0.211–42.624	0.417
*Staphylococcus aureus* detection	3.000	0.469–19.177	0.246
Mitral valve endocarditis	3.056	0.475–19.657	0.240
Severe valve regurgitation	2.476	0.428–14.342	0.312

*APT* antiplatelet therapy, *IVT* intravenous thrombolysis, *OAC* oral anticoagulation, *MRI* magnetic resonance imaging, *mRS* Modified Rankin Scale

**Table 4:** Intracranial hemorrhage, multivariate logistic regression analysis

	OR	95% CI	P-value
Female	0.188	0.020–1.127	0.093
Hospital stay	0.935	0.808–1.018	0.234
Thrombocytopenia	6.501	1.122–49.06	0.045*
IVT	5.591	0.307–252.0	0.289
*Staphylococcus aureus* detection	1.976	0.240–14.64	0.502

*IVT* intravenous thrombolysis

## Discussion

This single center study describes risk factors for intracranial hemorrhage in IE patients and outcome of patients with intracranial hemorrhage compared to patients with ischemic stroke. Hemorrhage occurred in all patients either before antibiotic therapy was started or in the first 9 days of treatment. Only one of these patients with intracranial hemorrhage showed infection control at time of intracranial hemorrhage. The risk of hemorrhagic as well as ischemic complications in IE is known to be elevated before treatment or in the first three weeks after treatment with the highest risk in week one [3].

All patients with intracranial hemorrhage had received (oral) anticoagulation, APT, IVT or had thrombocytopenia. However, among these factors, we identified only thrombocytopenia as a risk factor for intracerebral hemorrhage. In line with our study, thrombocytopenia as a risk factor for intracranial hemorrhage in IE has been described before [7]. In contrast to Garcia-Cabera et al. [3], we could not confirm any association between OAC intake and intracranial hemorrhage in IE. However, the group of patients with intracranial hemorrhage in our study was small and maybe therefore the data for OAC not conclusive. Data regarding IE and APT as risk for intracranial hemorrhage are inconclusive. Our data did not provide evidence of either an increased risk or a decreased risk of intracranial hemorrhage in patients with IE taking APT. Several studies demonstrate that APT is beneficial in treatment of IE to prevent related embolic events. A meta-analysis involving 5400 patients showed that acetyl-salicylic acid was associated with a significant decrease in major systemic embolism with the risk of intracranial hemorrhage tending to decrease [9]. In contrast, two studies by Chan et al. found that APT in IE was associated with an increased risk of intracranial hemorrhage, although not significantly [10–12]. Other studies showed no benefit, but as in our study also no elevated risk of hemorrhagic stroke [12–14].

In addition, we identified *S. aureus* as a risk factor for intracranial hemorrhage. *S. aureus* as a risk factor for embolism in IE is well-known, but to our knowledge as a risk factor for intracranial hemorrhage not reported [3]. Salaun et al. [7] detected other risk factors associated with intracranial hemorrhage in IE such as severe valve regurgitation and presence of IIA. In contrast, we did not identify severe valve regurgitation as a risk factor and only one of our patients with intracranial hemorrhage had an IIA. Nevertheless, the cohort in this study differed from our cohort in several aspects. For example, the patients in our study were slightly older and the proportion of men was higher. Thus, the differences in patient characteristics may contribute to the different findings. In contrast to our data, cerebral microbleeds seem to predict impending intracranial hemorrhage in IE in one study [15]. However, with 8 patients who developed intracranial hemorrhage, this study is relatively small and further data are needed to investigate the role of microbleeds as a risk factor for intracranial hemorrhage. Nevertheless, patients with microbleeds did not have a higher in-hospital mortality and estimated 1-year major adverse event rate in another study [16]. Furthermore, no patients with microbleeds developed postoperative intracranial hemorrhage after cardiac surgery [17].

IE patients with neurological complications have a worse clinical outcome. In line with other studies, we found an increased in-hospital mortality of 63.6% in patients with intracranial hemorrhage [3, 18]. In contrast to our results, Salaun et al. found a mortality of 24.6% within 2.3 months of follow-up in IE patients (n = 963) in general, but they did not find an increased mortality comparing IE patients with and without ICH [7]. However, as already mentioned, the cohort differed from ours. In our study, more patients with intracranial hemorrhage were detected with *S. aureus*. *S. aureus* is known to be associated with increased mortality. In addition, mortality rate in IE with intracranial hemorrhage seems to be related to the cause of intracranial hemorrhage. Mortality rate is 14% in patients with ruptured mycotic aneurysm, 15% in patients with HT and 45% in hemorrhage of undetermined etiology [7]. In our study, we had only 1 patient (9%) with IIA compared to 32.4% in the previously published cohort. Furthermore, Salaun et al. did not specify the criteria for HT, so it remains unclear whether patients with HI1 and HI2 were also included in the group with intracranial hemorrhage in the previously published study. We only considered PH1 and PH2 as relevant HT.

27.1% of all patients had a favorable outcome (mRS 0–2) on discharge in our study. As previously published, the patients with intracranial hemorrhage did not have a lower probability of favorable neurological outcome [19].

IE therapy is based on two major components, antibiotic therapy, and cardiac surgery, if indicated. The question of adequate timing of surgery arises with every stroke patient with IE, to avoid intracranial hemorrhage during surgery, but also to prevent further embolism or HF due to delayed surgery. The aim of surgery is radical resection of infected tissue, valve replacement and elimination of the source of embolism. Surgery is indicated in > 50% of IE patients [20]. Whenever cardiac surgery is indicated, it should be performed in a timely manner. However, in stroke patients it is often postponed. Patients with ischemic stroke or intracranial hemorrhage are at increased risk of (additional) intracranial hemorrhagic complications because of the inevitable use of high‐dose systemic anticoagulation during cardiopulmonary bypass.

In our study, cardiac surgery was performed in only 27.3% patients with intracranial hemorrhage and 43.2% patients with ischemic stroke. Overall, 15.7% new ischemic strokes occurred after valve reconstruction, whereas no new intracranial hemorrhage was observed. Surgery was performed 90 ± 92.7 days after admission in patients with intracranial hemorrhage and earlier, 43.14 ± 50.3 days, in patients with ischemic stroke. The low rate of cardiac surgery in the intracranial hemorrhage group is probably related to the early death of most of these patients within the first two weeks (5 from 7).

Although stroke is an independent risk factor for postoperative death in IE, it is not a contraindication for cardiac surgery in these patients unless there is extensive stroke or intracranial hemorrhage [2]. Due to lack of randomized controlled trials, recommendations for timing of cardiac surgery in stroke patients with IE are mainly based on single-center experiences and observational studies [2]. According to the ESC guideline, risk of post- operative neurological deterioration is low after silent cerebral emboli or transient ischemic attack, and surgery should be performed without delay [21]. For major ischemic stroke (2020 ACC/AHA) or intracranial hemorrhage (2015 ESC and 2020 ACC/AHA guidelines) delayed surgery > 4 weeks is recommended due to higher risk for earlier surgery. A multidisciplinary approach consisting of neurologists, neuroradiologists, cardiologists and cardiac surgeons is essential to define the optimal timing of cardiac surgery in these patients.

The limitations of our study are the single center and the retrospective design with all well-known limitations. Due to the rarity of the disease, the group of patients with patients with IE and intracranial hemorrhage was relatively small. In addition, at a university hospital, IE may be overrepresented as cause of stroke. For evaluation of HT, the ECASS II classification was used with PH1 and PH2 considered as relevant HT [8]. In our study, besides PH2, which is known to be associated with early deterioration, increased mortality, and disability in patients without IE, we also defined PH1 as relevant intracranial hemorrhage to include and assess all parenchymal hemorrhages in patients with HT and IE.

## Conclusion

To conclude, this single center study characterizes stroke patients with IE and raises awareness of important factors that contribute to IE complications. The in-hospital mortality is increased in patients with intracranial hemorrhage in our study. Beside *S. aureus* detection, we identified thrombocytopenia as a major risk factor for intracranial hemorrhage.

## Data Availability

The datasets used and/or analyzed during the current study are available from the corresponding author on reasonable request.

## Abbreviations

APT: Antiplatelet therapy
ECASS: European Cooperative Acute Stroke Study
HF: Heart failure
HI: Hemorrhagic infarction
HT: Hemorrhagic transformation of ischemic stroke
IE: Infective endocarditis
IIA: Infectious intracranial aneurysm
ICH: Intracerebral hemorrhage
IVT: Intravenous thrombolysis
MRI: Magnetic resonance imaging
mRS: Modified Rankin Scale
OAC: Oral anticoagulant
PH: Parenchymatous hematoma
SAH: Subarachnoid hemorrhage
S. aureus: Staphylococcus aureus
